# pH-triggered CS@ZnO_2_ nanocomposites: Self-activated ROS generation for efficient bacterial eradication

**DOI:** 10.3389/fbioe.2025.1608188

**Published:** 2025-05-20

**Authors:** Yu Zhang, Jun Liu, Sha Li, Jinhua Zhou, Jiushan Liu, Yan Huang

**Affiliations:** Department of Emergency, The Fourth Hospital of Changsha (Integrated Traditional Chinese and Western Medicine Hospital of Changsha), Changsha, Hunan, China

**Keywords:** chitosan, zinc peroxide, self-supply H_2_O_2_, reactive oxygen species, antibacterial

## Abstract

Functional nanomaterials based on reactive oxygen species (ROS) have attracted considerable attention in the treatment of bacterial infections, owing to their high sterilization efficiency and low tendency to induce drug resistance. Natural polymers, known for their excellent biocompatibility, have been widely used in the development of antibacterial dressings. In this study, chitosan-zinc peroxide composite dressing (CS@ZnO_2_) was synthesized using zinc acetate and chitosan as primary raw materials, and comprehensive characterizations were performed. Under the slightly acidic conditions of bacterial infections, CS@ZnO_2_ could self-decompose to release H_2_O_2_ and produce large amount of ROS, which would cause damage to bacteria. The *in vitro* antibacterial properties of CS@ZnO_2_ were investigated using *Escherichia coli* (*E*. *coli*) and *Staphylococcus aureus* (*S*. *aureus*) as representative pathogens. The results demonstrated that CS@ZnO_2_ exhibited potent antibacterial efficacy against both *S*. *aureus* and *E*. *coli*. This research provides an important theoretical foundation and technical support for the development of novel antibacterial materials, and has the potential to improve the efficacy of treatments for bacterial infections in the future.

## 1 Introduction

Bacterial infection is a major threat to human health. The discovery and development of antibiotics in the 20th century revolutionized medicine by drastically reducing the fatality rates associated with bacterial infectious diseases (Davies and Davies, 2010; Cook and Wright, 2022). However, the widespread and sometimes inappropriate use of antibiotics has led to an alarming rise in bacterial drug resistance. Bacteria have evolved mechanisms to survive exposure to antibiotics, rendering many traditional treatments less effective or even obsolete (Theuretzbacher et al., 2023). The growing prevalence of resistant strains necessitates the exploration of new antibacterial agents. In response to this challenge, researchers are investigating innovative materials and therapies that can combat bacteria in novel ways. Nanomaterials, including precious metals like silver and gold, non-metallic compounds, and antibacterial hydrogels, have shown remarkable promise due to their unique properties. These materials can disrupt bacterial cell walls, inhibit bacterial growth, and prevent biofilm formation, making them highly effective against both drug-sensitive and resistant strains (Xin et al., 2019; Makabenta et al., 2021; Stojanov et al., 2022; Chen et al., 2024; Yang et al., 2024). Although nano-antibacterial agents provide new strategies for combating bacterial infections, developing efficient and safe nano-antibacterial agents remains an arduous task.

Among the numerous emerging antibacterial strategies, the controllable generation of reactive oxygen species (ROS) for combating bacteria is attracting increasing attention. One of the key advantages of using ROS-based approaches is that they generally do not induce drug resistance, a significant concern with traditional antibiotics. This is because ROS attack multiple cellular targets simultaneously, making it difficult for bacteria to develop resistance mechanisms (Xie et al., 2020; Bi et al., 2022; Cao et al., 2022; Hwang et al., 2022; Sun et al., 2024). Since bacteria possess an endogenous antioxidant defense system to detoxify abnormal external oxidants (Dong and Wang, 2023). Hence, the prerequisite for ROS to be effective is to ensure that their content exceeds the bacterial tolerance threshold. To achieve the desired antibacterial effect, developing nanomaterials that can generate ROS efficiently is of paramount importance. Nanomaterials offer several advantages in this context. Their high surface area-to-volume ratio allows for enhanced reactivity, enabling more efficient production of ROS. Additionally, nanomaterials can be engineered to respond to specific environmental cues, such as pH changes, thereby providing targeted and controlled release of ROS directly at the site of infection.

Metal peroxides are efficient alternative sources of H_2_O_2_, and their physicochemical properties are more stable than those of H_2_O_2_ (Elbahri et al., 2017; Bai et al., 2022; Liu et al., 2022). They are composed of metal ions and peroxide groups and can reversibly release H_2_O_2_ in an acidic environment (Lin et al., 2019; Zhang et al., 2022). In recent years, nanomaterials have received attention due to their significant effects on antibacterial applications. Nevertheless, these materials still face challenges such as stability, preparation difficulty, and biosafety in practical applications. To solve these problems, this study aimed to synthesize a metal-based nanomaterial and investigate its antibacterial performance. The specific research contents are as follows: A chitosan-zinc peroxide composite dressing (CS@ZnO_2_) was prepared using zinc acetate, hydrogen peroxide, sodium hydroxide, and chitosan as raw materials. Through the characterization and analysis of the synthesized CS@ZnO_2_, the structural characteristics and performance parameters were elucidated. Then, *in vitro* antibacterial experiments against *E. coli* (*Escherichia coli*) and *Staphylococcus aureus* (*Staphylococcus aureus*) were conducted to validate the antibacterial performance of CS@ZnO_2_.

## 2 Experimental section

### 2.1 Preparation of ZnO_2_

0.5 g of zinc acetate dihydrate was dissolved in 20 mL of 5% sodium hydroxide solution. Then, 5 mL of 30% hydrogen peroxide was slowly added under continuous stirring, and the mixture was stirred for another 0.5 h. The resulting precipitate was centrifuged and washed five times with deionized water. The precipitate was then dried in an oven at 60°C. Finally, the ZnO_2_ product was ground into a fine powder using an agate mortar.

### 2.2 Preparation of CS@ZnO_2_

1 g of chitosan was dissolved in 20 mL of 2% acetic acid solution. Then, 10 mg of zinc acetate dihydrate was slowly added under continuous stirring, and the mixture was stirred for another 0.5 h. The resulting mixture was then freeze-dried to obtain chitosan-zinc ion complexes. Next, 25 mL of 5% sodium hydroxide solution was added to the chitosan-zinc ion complexes, and then 5 mL of 30% hydrogen peroxide was slowly added and reacted for a period. The mixture was then washed with deionized water to neutral pH. The CS@ZnO_2_ was obtained.

### 2.3 Characterization

Scanning electron microscopy (SEM) images were obtained from a Sigma 300 field emission scanning electron microscope (Carl Zeiss AG, Germany). Ultraviolet-visible (UV-vis) spectra were acquired on a UV-2450 spectrophotometer (Shimadzu Corporation, Japan). Fourier transform infrared (FT-IR) spectra were recorded via a Nicolet IS 5 Fourier transform infrared spectrometer (Thermo Fisher Scientific Inc., United States). X-ray diffraction (XRD) spectra were obtained from an Ultima IV X-ray diffractometer (Rigaku Holdings Corporation, Japan). X-ray photoelectron spectroscopy (XPS) was obtained by Thermo Scientific K-Alpha XPS (Thermo Fisher Scientific Inc., United States). Inductively coupled plasma mass spectrometry (ICP-MS) was obtained by Agilent 7,700x ICP-MS (Agilent Technologies Inc., United States). Confocal fluorescence imaging was performed using Leica TCS SP8 confocal luminescence microscope (Leica Microsystems Inc., Germany).

### 2.4 Characterization of peroxide groups

The peroxides groups of CS@ZnO_2_ were verified by a KMnO_4_ colorimetric assay. Briefly, CS@ZnO_2_ was added to KMnO_4_ solution (0.4 mM in 1 mM H_2_SO_4_). After incubation for 1 min, the color change of the solution was observed and the UV-vis absorption spectra were recorded immediately.

The production of ROS was confirmed using the 3,3′,5,5′-tetramethylbenzidine (TMB) colorimetric assay. Briefly, CS@ZnO_2_ was added to 0.5 mM TMB solutions at different pH values (pH 3, 5, 7, and 9). After incubation for 30 min at 37°C, the color change of the solution was observed and the UV-vis absorption spectra were recorded immediately.

Furthermore, titanium oxysulfate (TiOSO_4_) was selected as an indicator for H_2_O_2_ generation. TiOSO_4_ reacts with H_2_O_2_ to form a primrose yellow titanium peroxide complex (Ti(IV)O_2_^2+^), which exhibits UV-vis absorbance at 405 nm. Specifically, CS@ZnO_2_ was incubated in a pH five buffer solution for 2 h. Then, 0.5 mL of TiOSO_4_ (0.03 M) solution was added to 0.5 mL of the supernatant, followed by further incubation for 10 min to produce the yellow Ti(IV)O_2_^2+^ precipitate. Ti(IV)O_2_^2+^ precipitate was collected by centrifugation at 13,000 rpm for 5 min and re-dissolved in 1 mL H_2_SO_4_ (1 M) solution, and the UV-vis absorption spectra were recorded immediately.

### 2.5 Characterization of the liquid absorption performance of CS@ZnO_2_

The synthesized CS@ZnO_2_ was dried, and their mass was measured. Digital photographs of the dried samples were also taken for documentation. Subsequently, the CS@ZnO_2_ was subjected to compression under an external force, and the morphological changes were observed and recorded. Finally, the CS@ZnO_2_ were reintroduced into pure water, and the weight change after water absorption was measured.

### 2.6 Bacterial culture

*Escherichia coli* ATCC25922 (*E*. *coli*) and *S. aureus* ATCC6538 (*S*. *aureus*) were selected as model Gram-negative and Gram-positive bacteria, respectively. Bacterial cells were grown overnight in LB medium (Luria-Bertani broth, Lennox modification) at 37°C and then harvested at the exponential growth phase via centrifugation. *E*. *coli* or *S*. *aureus* cells were washed twice to remove residual macromolecules and other growth medium constituents and re-suspended in sterile saline solution (0.9% NaCl). Bacterial suspensions with an optical density at 600 nm (OD600 nm) of 0.1 were used for the subsequent experiments.

### 2.7 Biocompatibility evaluation

Fresh human blood (ethical approval no. CSSDSYY-YXLL-SC-2023–03–20) was collected in heparinized tubes and centrifuged (3,000 rpm, 10 min) to isolate erythrocytes. Subsequently, the erythrocyte suspension (2% v/v) was incubated with CS@ZnO_2_ solutions at 37°C for 4 h. Positive control (pure water) and negative control (0.9% NaCl solution) were included. After incubation, the samples were centrifuged again at 3,000 rpm for 10 min, and the absorbance of the supernatant was measured at a wavelength of 540 nm. The hemolysis ratio was calculated as follows:

$$\text{Hemolysis ratio }\left(\%\right)=\frac{{\text{OD}}_{\text{sample}}-{\text{OD}}_{\text{negative}}}{{\text{OD}}_{\text{positive}}-{\text{OD}}_{\text{negative}}}\times 100\%$$

### 2.8 Antibacterial assay

CS@ZnO_2_ was added to the bacteria suspensions, and then the mixture was incubated at 37°C with shaking at 200 rpm for 24 h. The antibacterial property of CS@ZnO_2_ was determined through the plate spreading experiment. Following the antibacterial experiments, the bacteria were harvested via centrifugation and subsequently washed with phosphate-buffered saline (PBS). According to the instructions of the bacterial viability assay kit (Beyotime Biotechnology, China), the samples were incubated with N,N-dimethylaniline N-oxide (DMAO) and propidium iodide (PI). In this process, viable bacteria were labeled with DMAO, producing green fluorescence, while non-viable bacteria were additionally stained with PI, resulting in red fluorescence emission. To distinguish between live and dead cells, confocal laser scanning microscopy was employed for imaging analysis.

To evaluate the production of reactive oxygen species (ROS) induced by CS@ZnO_2_, the 2′,7′-dichlorodihydrofluorescein diacetate (DCFH-DA) assay was employed. In this process, the CS@ZnO_2_ treated bacteria were incubated with DCFH-DA following the protocol provided in the ROS assay kit (Beyotime Biotechnology, China). Notably, DCFH-DA lacks fluorescence and can permeate bacterial cell membranes freely. Once inside the cells, it undergoes hydrolysis mediated by intracellular esterases, leading to the formation of DCFH, a compound that cannot exit the cells. Subsequently, intracellular ROS oxidize the non-fluorescent DCFH into fluorescent DCF. The intensity of DCF fluorescence serves as an indicator of the concentration of intracellular ROS.

## 3 Results and discussion

### 3.1 Antibacterial design strategies

The CS@ZnO_2_ nanocomposite, designed in this study, demonstrates remarkable stability under neutral conditions due to its chitosan shell. Chitosan, a natural polysaccharide derived from chitin, is known for its biocompatibility and biodegradability. This protective layer ensures that the ZnO_2_ remains intact and stable in physiological environments, preventing premature release or degradation. However, when exposed to slightly acidic conditions, such as those found in areas of bacterial infection, the chitosan shell exhibits acid-responsive dissociation (Hageman et al., 2024). This property is crucial because many pathogenic bacteria create a microenvironment with lower pH levels as they proliferate. Upon encountering these acidic conditions, the chitosan shell begins to break down, releasing the encapsulated ZnO_2_ *in situ*. Once released, ZnO_2_ undergoes self-decomposition under acidic conditions. This process leads to the generation of H_2_O_2_ (Elbahri et al., 2017). H_2_O_2_ is a potent antimicrobial agent that can generate ROS. These ROS cause irreversible damage to bacterial cellular components, including proteins and DNA, effectively eliminating the pathogens without inducing drug resistance (Zhu et al., 2021; Jannesari et al., 2023; Wang et al., 2023).

### 3.2 Characterization of CS@ZnO_2_

**FIGURE 1:**
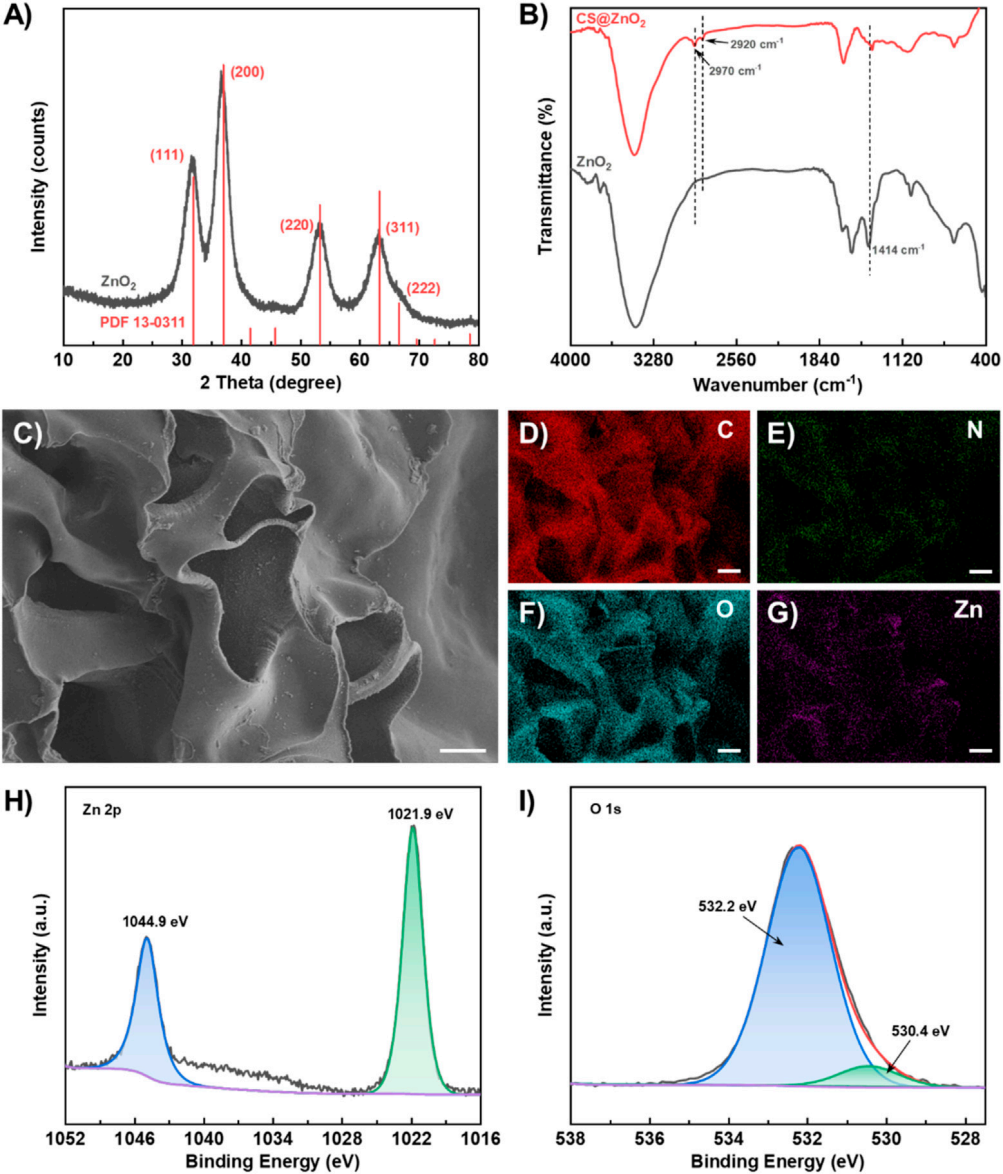
Characterization of CS@ZnO_2_. **(A)** XRD pattern, **(B)** FT-IR spectra, **(C)** SEM image, **(D–G)** Elemental mapping images of C, O, N, and Zn, **(H)** Zn 2p spectrum and **(I)** O 1s spectrum.

The synthesis of ZnO_2_ was characterized using XRD and the result is shown in Figure 1A. The diffraction peaks at 2θ = 31.9°, 37.0°, 53.2°, 63.3°, and 66.6° correspond to the (111) (200) (220) (311), and (222) of ZnO_2_ (JCPDS card no. 13–0,311) respectively. The calculated d-values for these planes (2.80 Å, 2.43 Å, 1.72 Å, 1.47 Å and 1.40 Å) are in good agreement with the reference data. CS@ZnO_2_ was further characterized using infrared absorption spectroscopy (FT-IR). As shown in Figure 1B, CS@ZnO_2_ exhibits a characteristic peroxide (-O_2_^2-^) ion O-O bond absorption peak at 1,414 cm^–1^ (Huang et al., 2022). Additionally, there are obvious characteristic absorption peaks at 2,920 cm^–1^ and 2,970 cm^–1^, which are attributed to the asymmetric and symmetric stretching vibration peaks of -CH_2_- of chitosan, respectively (Ding et al., 2022; García Cambón et al., 2023). The morphology of CS@ZnO_2_ was characterized using scanning electron microscopy (SEM), and the results are shown in Figure 1C. The synthesized CS@ZnO_2_ exhibit a porous structure, which enhances their capacity for liquid absorption. Additionally, granular substances have been observed to be embedded in the chitosan substrate. The elemental mapping analysis via SEM coupled with energy-dispersive X-ray spectroscopy (EDS) demonstrates a homogeneous spatial distribution of C, N, O, and Zn throughout the CS@ZnO_2_ (Figures 1D–G). Quantitative analysis reveals that the atomic percentages of these elements are 60.04%, 10.32%, 29.13% and 0.51%, respectively (Supplementary Figure S1). The X-ray photoelectron spectroscopy (XPS) full-scan and high-resolution spectra were collected to investigate the chemical states of the CS@ZnO_2_. As shown in Supplementary Figure S2, the XPS survey spectrum confirms the coexistence of C, N, O, and Zn, with their characteristic peaks appearing at binding energies of 285.7 eV, 398.9 eV, 532.2 eV and 1,021.9 eV, respectively. The Zn 2p spectrum (Figure 1H) exhibits spin-orbit splitting peaks at 1,021.9 eV (2p_3/2_) and 1,044.9 eV (2p_1/2_), which is consistent with the characteristic of Zn(II) oxide (Lin et al., 2019; Zhang et al., 2022). High-resolution scans of the O 1s region (Figure 1I) were deconvoluted into two distinct components using Gaussian-Lorentzian fitting. The dominant peak at 532.2 eV corresponds to O_2_^2-^, while the minor peaks at 530.4 eV is attributed to O^2-^, indicating the presence of peroxide groups (Lin et al., 2019; Zhang et al., 2022). Quantitative analysis based on peak area ratios indicates that the O_2_^2-^ accounts for 92.2% of the total O 1s content. The above results indicate that the CS@ZnO_2_ was successfully synthesized.

### 3.3 Acid-induced CS@ZnO_2_ generates ROS

**FIGURE 2:**
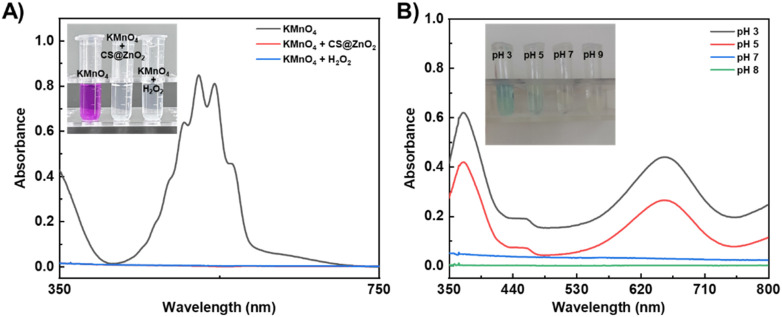
Acid-responsive self-decomposition properties of CS@ZnO_2_. **(A)** Absorption spectra and photograph (inset) of KMnO_4_ (in 1 M H_2_SO_4_) solution after incubation with CS@ZnO_2_ or H_2_O_2_. **(B)** Absorption spectra and photograph (inset) of TMB solution after incubation with CS@ZnO_2_ at different pH buffers.

The classic redox reaction between potassium permanganate and hydrogen peroxide was used to determine whether ZnO_2_ would self-decompose to release H_2_O_2_ under acidic conditions (Zhang et al., 2016; Sun et al., 2019). As expected (Figure 2A), the purple solution of acidic KMnO_4_ became colourless and the characteristic absorption peaks disappeared after the addition of H_2_O_2_ or ZnO_2_. This is because the H_2_O_2_ can reduce the purple MnO_4_^−^ to colourless Mn^2+^ in acidic media (Equation 1), while ZnO_2_ can self-decompose and release H_2_O_2_ under acidic conditions. These results further demonstrate the successful preparation of ZnO_2_ and the nature of its self-decomposition to release H_2_O_2_ under acidic conditions. Furthermore, the concentration of released H_2_O_2_ was quantitatively determined using the titanium oxysulfate colorimetric method (Zhang et al., 2018; Gong et al., 2021). Based on the standard curve constructed with the standard H_2_O_2_ concentrations (Supplementary Figure S3), the amount of released H_2_O_2_ was calculated to be 287.7 ± 54.3 µM.

$$2{\text{MnO}}_{4}^{-}+5H_{2}O_{2}+6H^{+}\to 2{\text{Mn}}^{2+}+5O_{2}↑+8H_{2}O$$ (1)

Taking advantage of the property that the colourless 3,3′,5,5′-tetramethylbenzidine (TMB) can be oxidized by ROS (such as OH^−^) to form a blue TMB oxide (ox-TMB) with a strong absorption peak at 652 nm (Song et al., 2019; Aham et al., 2025; Wang et al., 2025), the ability of acid-induced CS@ZnO_2_ to generate ROS was confirmed. As expected (Figure 2B), under weakly acidic conditions (pH 5), CS@ZnO_2_ turned the TMB solution blue (inset of Figure 2B), and showed a strong absorption peak at 652 nm. This phenomenon was attributed to the self-decomposition of CS@ZnO_2_ under weakly acidic conditions and the release of H_2_O_2_. Subsequently, H_2_O_2_ decomposed to produce ROS, such as OH^−^, which oxidized TMB to form the blue ox-TMB. In addition, the intensity of the absorption peak at 652 nm and the blue color of the solution increased with the acidity (e.g., pH 3), indicating that the acidic environment was conducive to the decomposition of ZnO_2_ in CS@ZnO_2_ and promoted the production of more ROS. Conversely, the absorbance and color of the TMB solution remained basically unchanged at pH 7, suggesting that CS@ZnO_2_ remained relatively stable under neutral conditions.

The above results indicate that the self-decomposition of ZnO_2_ in CS@ZnO_2_ can be activated under weak acidic conditions. By utilizing the acidic microenvironment (pH 5.0–6.0) associated with bacterial infection, the self-decomposition of ZnO_2_ in CS@ZnO_2_ can be triggered to release H_2_O_2_ and ROS on demand. CS@ZnO_2_ demonstrates significant potential for the treatment of bacterial infections.

Notably, research indicates that the released Zn^2+^ also exhibit antibacterial properties due to their significant inhibition of the active transport process in microbial cells, interference with amino acid metabolic pathways, and disruption of enzyme systems (Sirelkhatim et al., 2015). The Zn^2+^ concentration released from CS@ZnO_2_ under neutral (pH 7) and acidic (pH 5) conditions after 24 h of incubation was quantitatively characterized using inductively coupled plasma mass spectrometry (ICP-MS). The results demonstrated concentrations of released Zn^2+^ were 9.2 µM and 58.6 µM under neutral and acidic conditions, respectively. Previous studies have shown that bacteria have a certain tolerance to low concentrations of Zn^2+^, and only when the Zn^2+^ concentration reaches the millimolar level will it exhibit significant toxic effects on bacteria (Xiong and Jayaswal, 1998; Outten and O'Halloran, 2001; Reddy et al., 2007). Combined with the ICP-MS results, it can be inferred that the released Zn^2+^ does not contribute to the antibacterial performance of CS@ZnO_2_.

### 3.4 The liquid absorption performance of CS@ZnO_2_

Another important step in the management of bacterially infected wounds is “drainage,” which involves removing any fluid that has accumulated on the wound and directing it to an external dressing (Zerbe et al., 1996; Mellinghoff et al., 2019). This helps to remove bacteria from the wound and allows nutrients from the blood to be transported to the wound to promote healing (Williams and Barbul, 2012; Powers et al., 2016). Therefore, the absorption performance of the synthesized CS@ZnO_2_ was evaluated through a water absorption test.

**FIGURE 3:**
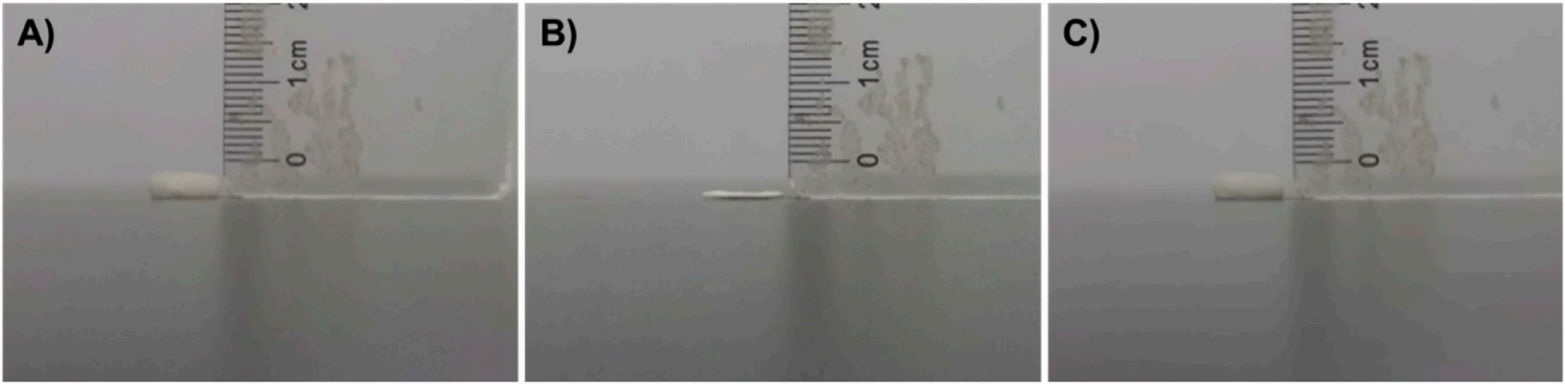
Characterization of CS@ZnO_2_ hydrogels. Photographs of the CS@ZnO_2_ in **(A)** the original state, **(B)** the force-compressed state, and **(C)** the shape-recovered state after water absorption.

As shown in Figure 3, CS@ZnO_2_ has a naturally fluffy appearance and a large volume. When subjected to external compression, the size and volume of CS@ZnO_2_ were significantly reduced (Figures 3A, B). However, after reabsorption of water, CS@ZnO_2_ can return to its original state (Figure 3C), indicating excellent liquid absorption and compression recovery properties. In addition, the water absorption capacity was assessed by measuring the mass of CS@ZnO_2_ before and after water absorption. The results show that CS@ZnO_2_ can absorb up to 27.2 times its dry weight (dry weight is 7.4 mg, wet weight is 208.8 mg), highlighting its superior liquid absorption performance and significant potential for medical applications.

### 3.5 The antibacterial properties of CS@ZnO_2_

To biocompatibility of CS@ZnO_2_ was investigated through hemolysis tests to verify its safety and potential value in biomedical applications. As shown in Supplementary Figure S4, the solution of the positive control group exhibited a distinct red coloration, whereas no visible color change was observed in either the negative control group or the CS@ZnO_2_ experimental group. Quantitative assessment of hemolytic activity revealed a remarkably low hemolysis rate of 1.16% ± 0.25% for the CS@ZnO_2_ group, demonstrating exceptional blood compatibility that complies with the stringent <5% safety criterion for biomedical devices outlined in ISO 10993–4.

**FIGURE 4:**
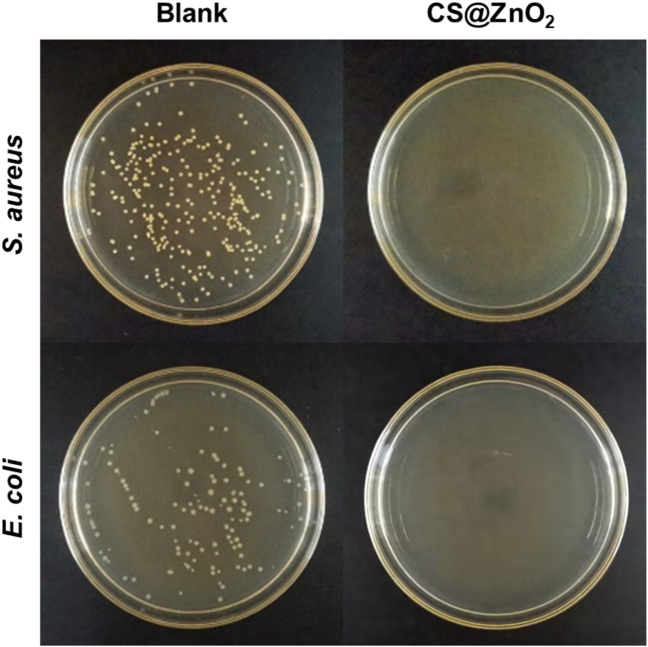
Antibacterial properties of CS@ZnO_2_ against *Staphylococcus aureus* and *Escherichia coli*.

*Staphylococcus aureus*, a typical Gram-positive bacterium, is known to cause many serious infections. *E*. *coli*, a typical Gram-negative bacterium, is also associated with numerous severe infections. In this experiment, the antimicrobial properties of CS@ZnO_2_ against both Gram-positive and Gram-negative bacteria were investigated, using *S*. *aureus* and *E*. *coli* as representatives, respectively. Specifically, CS@ZnO_2_ was co-incubated with the bacteria for 24 h, and the antibacterial effect was evaluated by the plate count method. As shown in Figure 4S *Aureus* exhibited significant colony formation in the control group, while no colonies were observed after incubation with CS@ZnO_2_. Similarly, *E*. *coli* demonstrated robust colony formation in the control group, and no colonies were observed after incubation with CS@ZnO_2_ either. These results indicate that CS@ZnO_2_ exhibits excellent antibacterial performance.

**FIGURE 5:**
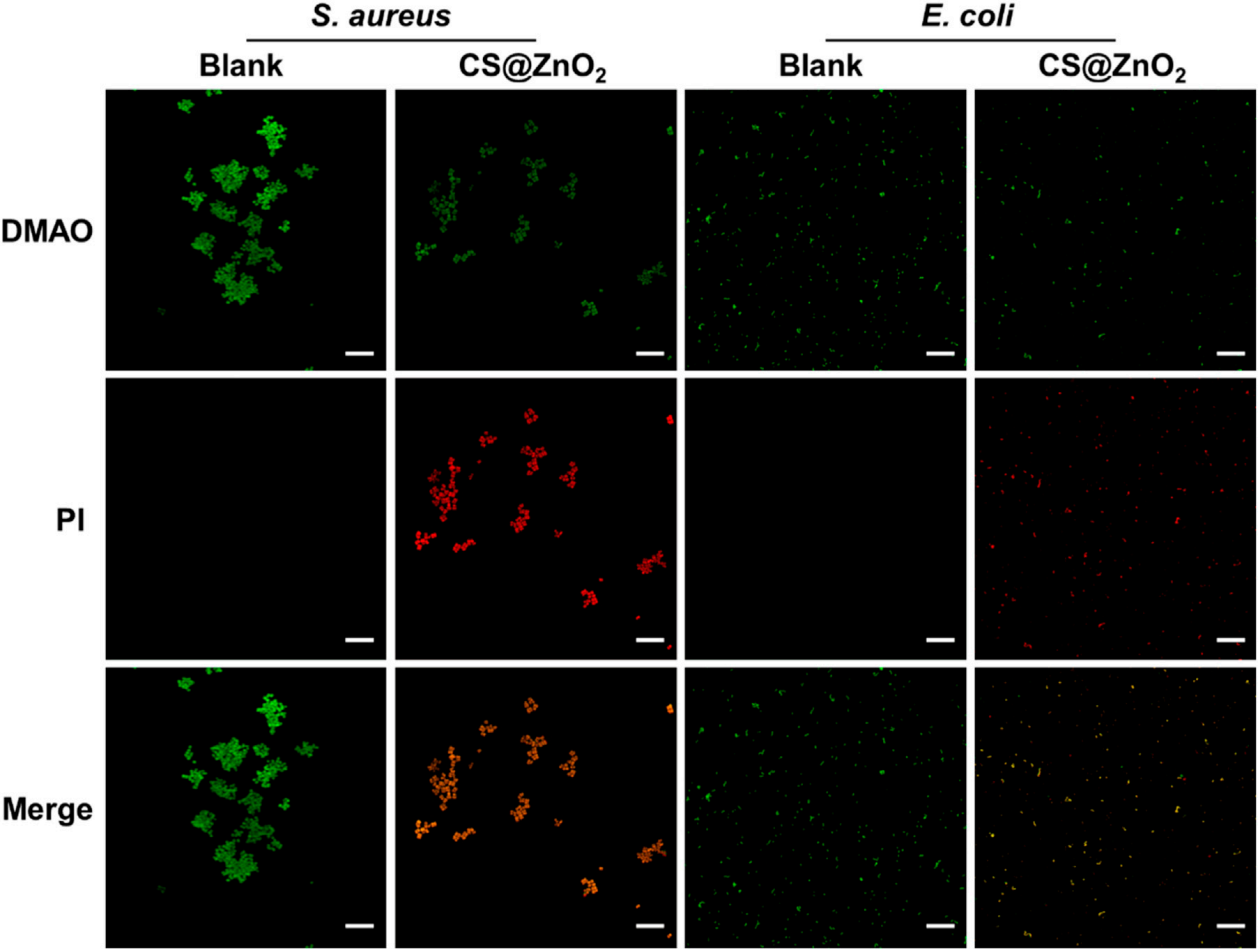
Confocal laser scanning microscopy images. CLSM images of *Staphylococcus aureus* and *Escherichia coli* after incubation with or without CS@ZnO_2_. Stained with DMAO (green) and PI (red), scale bars = 25 μm.

To further evaluate the antimicrobial efficacy of CS@ZnO_2_, bacterial viability was assessed through differential fluorescence staining. N,N-dimethylaniline N-oxide (DMAO), a green fluorescent nucleic acid dye, is capable of staining both live and dead bacteria due to its membrane-permeable properties. Propidium iodide (PI), in contrast, is a non-permeable fluorescent dye that cannot penetrate intact cell membranes of viable cells, making it ineffective for staining bacteria with undamaged membranes. However, in necrotic bacteria where membrane integrity is compromised, PI can enter the nucleus, bind to double-stranded DNA, intercalate into the DNA double helix, and form a PI-DNA complex, resulting in red fluorescence. When DMAO and PI are used in combination to detect the viability of bacteria, live bacteria with intact cell membranes exhibit only green fluorescence, whereas dead bacteria with damaged cell membranes display both green and red fluorescence. As shown in Figure 5, the blank group exhibited exclusively green fluorescence without any detectable red fluorescence, indicating that the bacteria were viable. In contrast, bacteria exposed to CS@ZnO_2_ exhibited both green fluorescence and red fluorescence, suggesting that the bacteria membranes had been compromised and the cells inactivated. These results demonstrate that CS@ZnO_2_ possesses significant antibacterial properties.

The generation of reactive oxygen species in bacteria after treatment with CS@ZnO_2_ was monitored using DCFH-DA. As shown in Supplementary Figure S5, compared with the untreated control group, bacteria exposed to CS@ZnO_2_ exhibited significantly enhanced fluorescence intensity, indicating that CS@ZnO_2_ can effectively stimulate the generation of ROS. The increase in ROS levels in the CS@ZnO_2_ treatment group may be due to the *in-situ* decomposition of ZnO_2_, which promotes the release of hydrogen peroxide and further enhances the generation of ROS. Such high concentrations of ROS damage bacterial membranes and intracellular components (such as DNA and proteins), ultimately leading to bacterial apoptosis.

## 4 Conclusion

In summary, CS@ZnO_2_ with acid-responsive properties has been successfully synthesized and exhibit significant antibacterial performance. The synthesized CS@ZnO_2_ remains stable under neutral conditions due to its chitosan shell, while it undergoes acid-responsive dissociation in the acidic microenvironment of bacterial infections. Following the dissociation of the chitosan shell, ZnO_2_ is released *in situ* and subsequently decomposes under acidic conditions to produce bactericidal H_2_O_2_. The decomposition of H_2_O_2_ irreversibly damages bacteria, thereby achieving effective antibacterial activity without inducing bacterial resistance. This study provides a theoretical foundation for the development of novel clinical nano-antimicrobial agents and offers a basis for their further application in combating bacterial infections.

## Data Availability

The original contributions presented in the study are included in the article/Supplementary Material, further inquiries can be directed to the corresponding author.
