# Supplementary material for: pH-triggered CS@ZnO2 nanocomposites: Self-activated ROS generation for efficient bacterial eradication
Source: Front Bioeng Biotechnol. 2025 May 20;13:1608188. doi: 10.3389/fbioe.2025.1608188 (PMC12129926; doi:10.3389/fbioe.2025.1608188)
Supplement: Supplementary file 1 [file DataSheet1.docx]

**Supporting Information**

pH-triggered CS@ZnO_2_ nanocomposites: self-activated ROS generation for efficient bacterial eradication

Yu Zhang^*^, Jun Liu, Sha Li, Jinhua Zhou, Jiushan Liu, Yan Huang

Department of Emergency, The Fourth Hospital of Changsha (Integrated Traditional Chinese and Western Medicine Hospital of Changsha), Changsha, Hunan, China

*** Correspondence:**

Corresponding Author: Yu Zhang

zhangydr@126.com


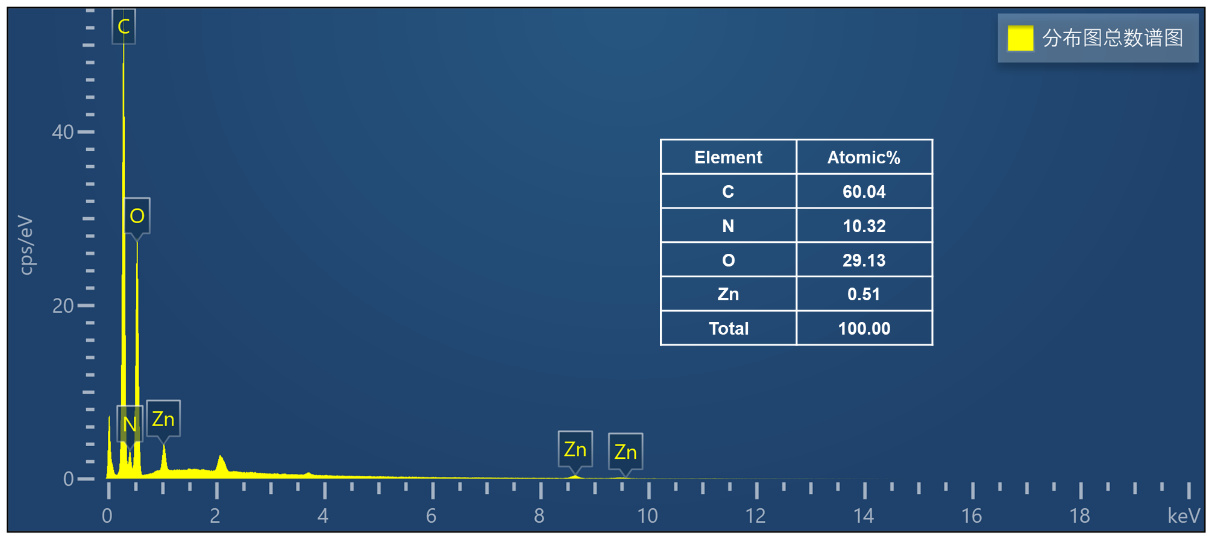


Fig. S1 SEM-EDX elemental spectra of CS@ZnO_2_.


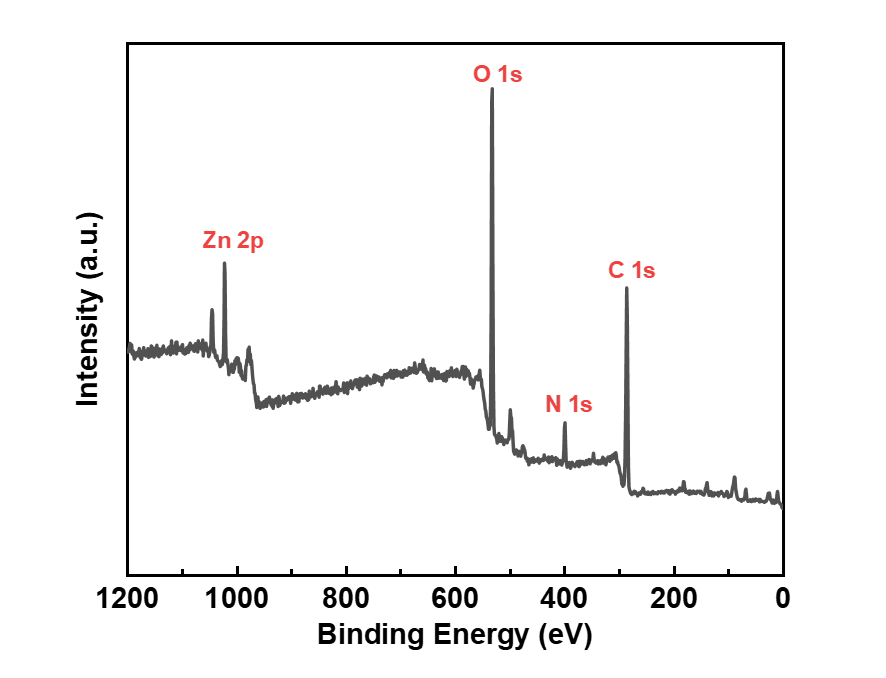


Fig. S2 XPS survey spectrum of CS@ZnO_2_.


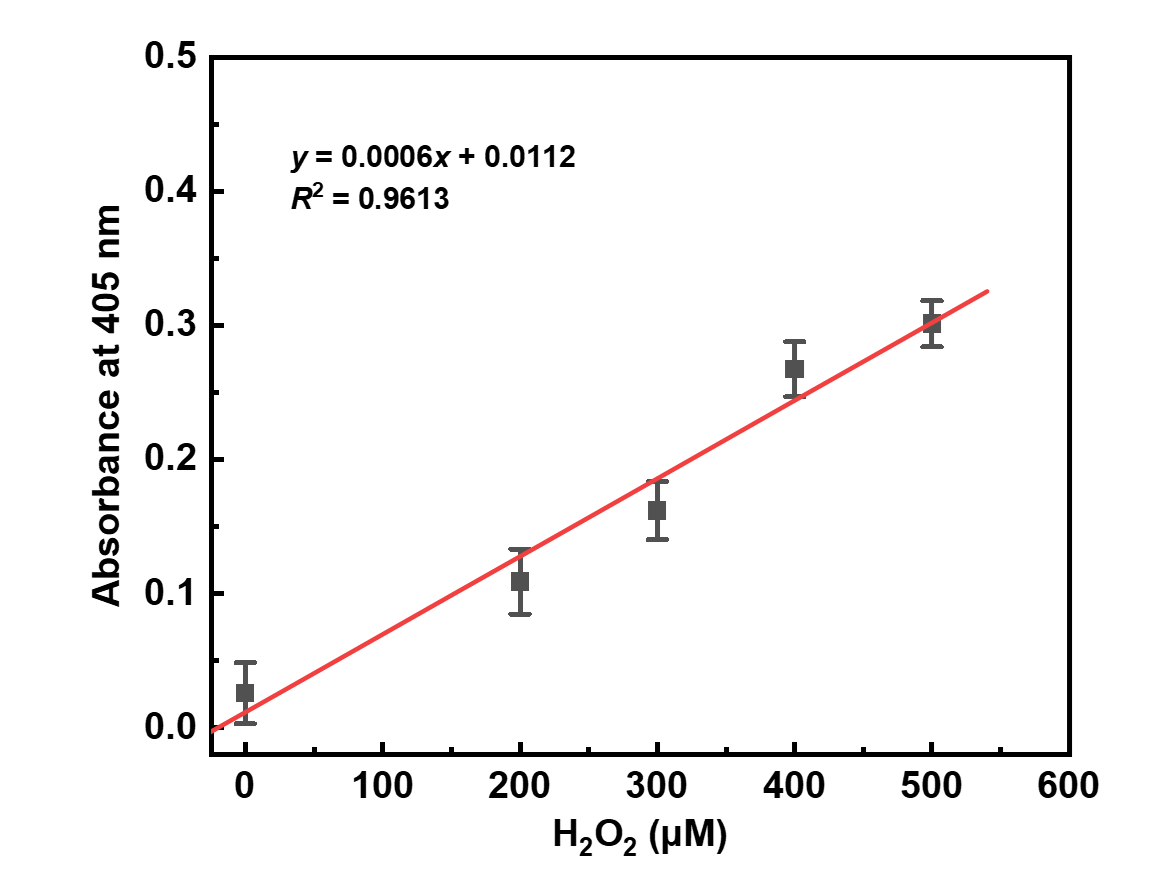


Fig. S3 Standard curve drawn from the reaction of known concentrations of H_2_O_2_ and TiOSO_4_


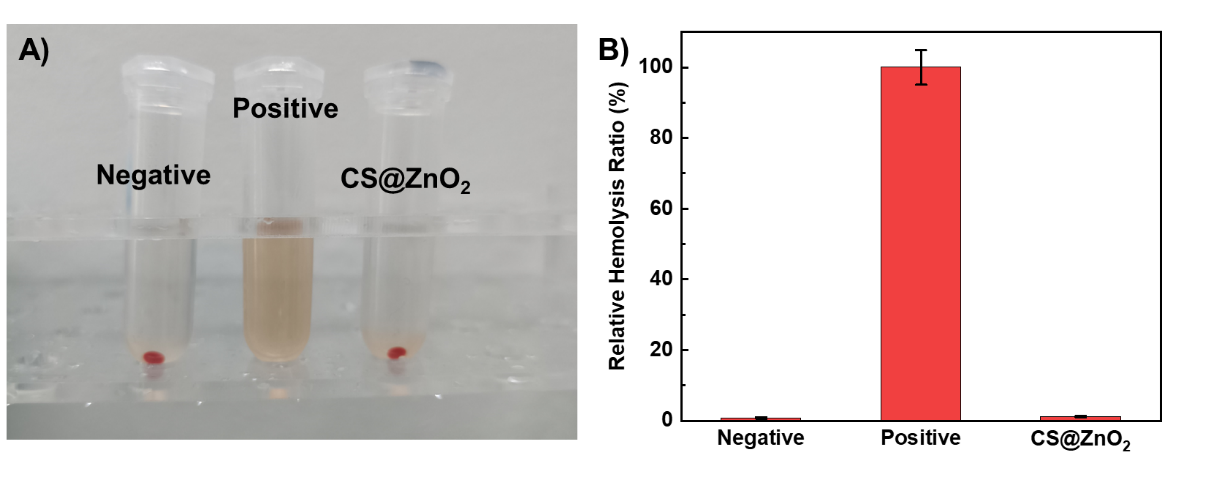


Fig. S4 (A) Digital image and (B) hemolysis ratio of the hemolytic test results.


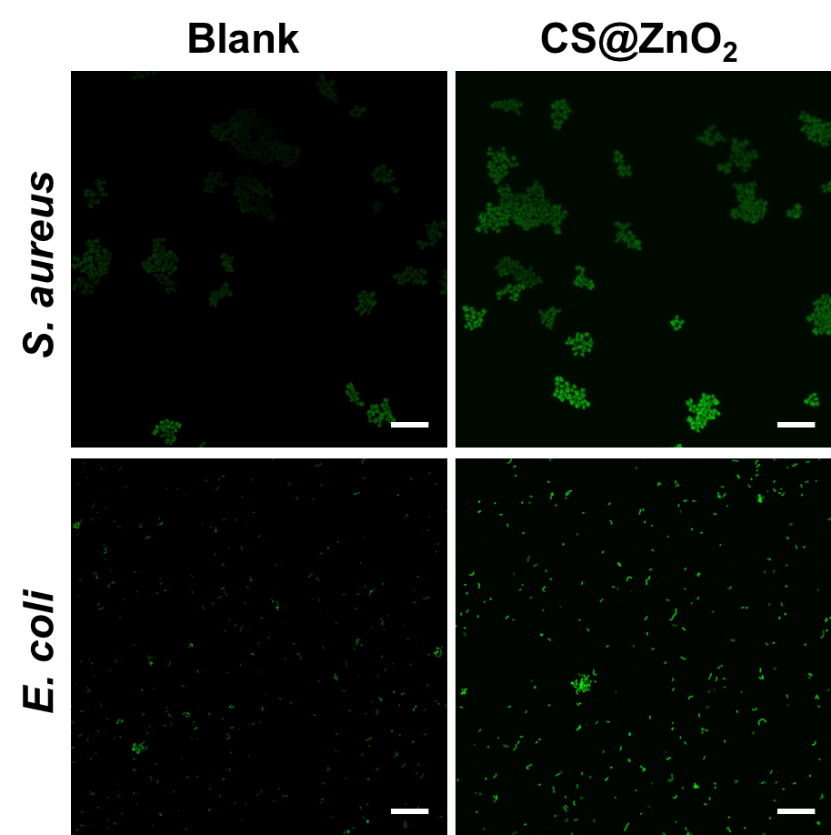


Fig S5 Confocal laser scanning microscopy images of the relative ROS level of *S*. *aureus* and *E*. *coli* after incubation with CS@ZnO_2_. scale bars = 25 μm.
